# A Metal-Based Receptor for Selective Coordination and Fluorescent Sensing of Chloride

**DOI:** 10.3390/molecules26082352

**Published:** 2021-04-18

**Authors:** Mauro Formica, Vieri Fusi, Daniele Paderni, Gianluca Ambrosi, Mario Inclán, Maria Paz Clares, Begoña Verdejo, Enrique García-España

**Affiliations:** 1 Department of Pure and Applied Sciences, University of Urbino “Carlo Bo”, Via della Stazione 4, 61029 Urbino, Italy; vieri.fusi@uniurb.it (V.F.); daniele.paderni@uniurb.it (D.P.); gianluca.ambrosi@uniurb.it (G.A.); 2 Institute of Molecular Sciences, University of Valencia, C/Catedrático José Beltrán 2, 46980 Paterna Valencia, Spain; M.Paz.Clares@uv.es (M.P.C.); begona.verdejo@uv.es (B.V.); enrique.garcia-es@uv.es (E.G.-E.)

**Keywords:** macrocyclic polyamine, metallo-receptor, fluorescence, chloride sensing

## Abstract

A scorpionate Zn^2+^ complex, constituted by a macrocyclic pyridinophane core attached to a pendant arm containing a fluorescent pyridyl-oxadiazole-phenyl unit (PyPD), has been shown to selectively recognize chloride anions, giving rise to changes in fluorescence emission that are clearly visible under a 365 nm UV lamp. This recognition event has been studied by means of absorption, fluorescence, and NMR spectroscopy, and it involves the intramolecular displacement of the PyPD unit by chloride anions. Moreover, since the chromophore is not removed from the system after the recognition event, the fluorescence can readily be restored by elimination of the bound chloride anion.

## 1. Introduction

Recognition of anionic species by fluorescent receptors has given rise to a great deal of interest in the last years, and several books and review articles discussing this chemistry have been published [1,2,3,4,5,6,7,8,9,10,11,12,13]. Many of the reported examples involve molecules with fluorescent organic components whose luminescence changes significantly upon their interaction with a target anion. In addition, a number of the receptor molecules used are metallo-receptors having Lewis acid metal sites as recognition points for the anionic guest species [14,15,16,17,18,19,20,21]. On the other hand, there are also different examples of the use of displacement assay methods in anion recognition [22,23,24,25]; namely, a chromophoric or fluorophoric anionic dye bound to the receptor is removed by a competing non-chromophoric anionic species, giving rise to a sharp change in its optical response. For example, evaluation of DNA intercalators by their competition with ethidium bromide or of G-quadruplex binders by thiazole orange are displacement assays very broadly employed in nucleic acid chemistry [26,27,28].

Chloride anion is an important target to monitor due to its role in many fields; its distribution in the biological environment means it often play critical roles such as, for example, in the interaction with hemoglobin, where the alteration of its concentration in plasma is associated with many pathologies [29,30,31].

For this reason, many sensor systems to detect chloride have been developed [32,33,34], many of which involve binding chloride by H-bonding or charge-charge interactions, while a few exploit the photochemical response of a metallo-receptor able to bind chloride [35,36,37,38].

**Figure 1:**
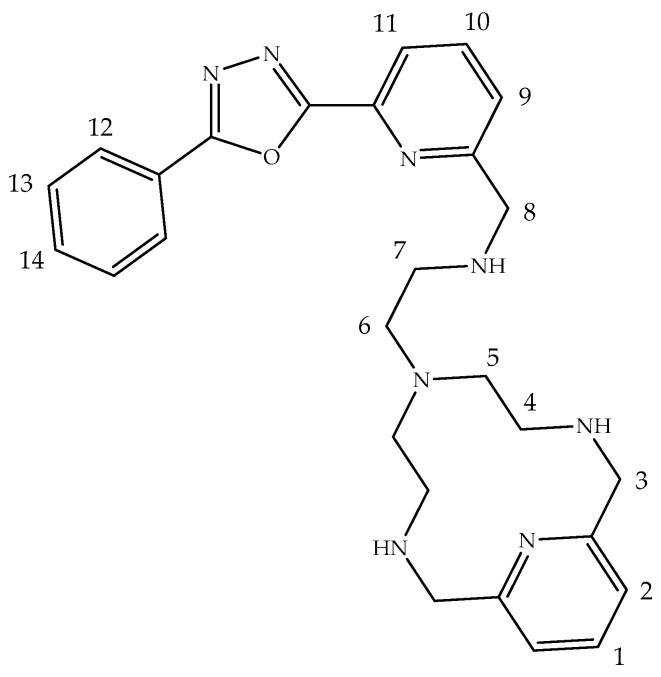
Structure of L with ^1^H-NMR labeling scheme.

Here, we report the case of a Zn^2+^ complex ([ZnL]^2+^) whose luminescence changes selectively upon binding of chloride anion due to a molecular reorganization which involves the removal of a bound pyridine nitrogen attached to an oxadiazole-phenyl group. The ligand is constituted by a macrocyclic pyridinophane core having a fluorescent pendant arm containing a secondary nitrogen atom linked through a methylene spacer to a pyridyl-oxadiazole-phenyl (PyPD) system (Figure 1). The Zn^2+^ complex can interact with the target analyte displacing the pyridine of the pyridyl-oxadiazole-phenyl system performing, thereby, a significant change in the fluorescence.

Since the chloride displaces the chromophore without removing it from the system, the fluorescence can be readily restored by precipitation of the chloride anions with, for example, silver cations. A similar kind of mechanism has been described—although without a Lewis acid metal site—for the recognition of glyphosate anions [39].

## 2. Results and Discussion

### 2.1. Spectrophotometric Studies

The free ligand is not emissive in CH_3_CN as it is totally quenched due to the PET effect of the aliphatic amine lone-pairs of the side arm [40,41]; however, the coordination of Zn^2+^ to give the [ZnL]^2+^ species inhibits the PET effect, giving rise to an intense fluorescence emission (Φ_f_ = 0.24) centered at 374 nm (λ_ex_ = 278 nm).

Considering the highly fluorescent response exhibited by the [ZnL]^2+^ species in CH_3_CN, a screening of its photochemical response to the addition of several anions was performed in such medium. The screened anions include F^−^, Cl^−^, Br^−^, I^−^, NO_3_^−^, HSO_4_^−^, H_2_PO_4_^−^, and CH_3_COO^−^ as their tetrabutylammonium salts. They were added in up to 5-fold excess to a CH_3_CN solution containing the preformed [ZnL](ClO_4_)_2_ complex (Figure S1, Supplementary Materials). Chloride has been the only anion able to affect the absorption, fluorescence, and ^1^H-NMR spectra. Similar experiments, performed in aqueous buffer pH = 7.4 solution, did not afford significant modifications although a little decrease in the emission was found by the addition of the chloride anion (Figure S2).

**Figure 2:**
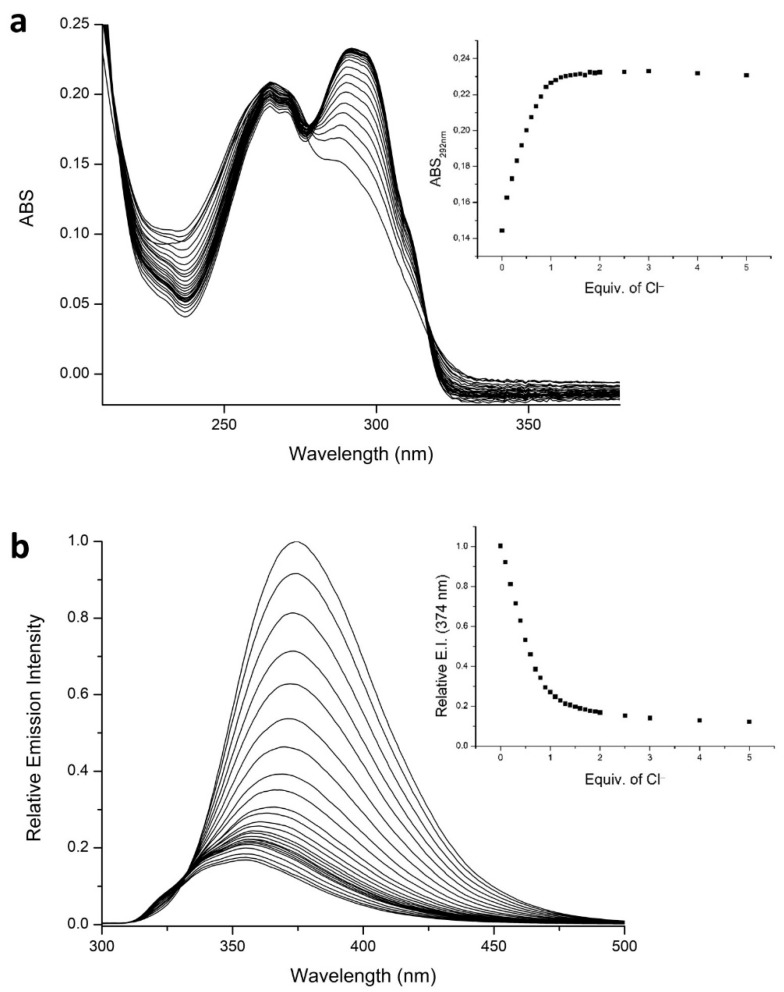
UV-Vis absorption (**a**) and fluorescence titration (**b**) of [ZnL]^2+^ species (1.35 × 10^−5^ mol dm^−3^; λ_ex_ = 278 nm) with Bu_4_NCl in CH_3_CN solution at 298 ± 0.1 K. Inset: trend of the absorption at 292 nm and of the emission intensity at 374 nm as a function of Cl^−^ added.

**Figure 3:**
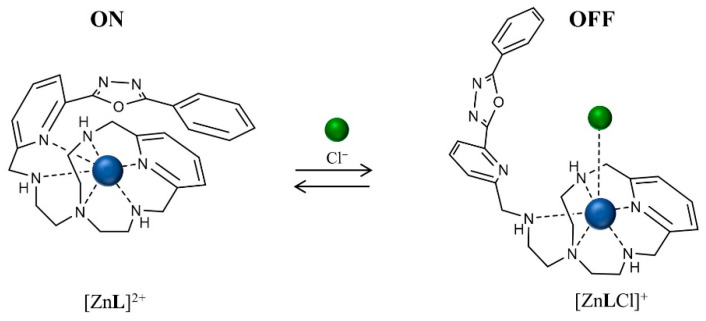
Proposed scheme for the formation of [ZnLCl]^+^ species.

The addition of chloride affects both the [ZnL]^2+^ absorption and fluorescence spectra (Figure 2). In the absorption spectra, a lower energy band centered at 290 nm appears. This absorption band can be safely ascribed to the PyPD chromophore not involved in metal coordination; in fact, the free ligand L shows an intense absorption band centered at 290 nm. The emission strongly decreases, although it is not fully quenched, and the emission band shifts toward higher energy; once again, this shift is in agreement with the emission properties of the ligand in which the PyPD is not involved in coordination [42] (Figure S3). The trends of both the absorption band at 292 nm and the emission at 374 nm, reach a plateau after the addition of one equivalent of chloride, highlighting that only one anion can be bound by the [ZnL]^2+^ species (see inset in Figure 2a,b), in agreement with the NMR experiments (vide infra). These results can be related, as mentioned above, to the replacement of the PyPD fragment from the coordination of Zn^2+^ by the chloride anion. This implies that the PyPD moiety is no longer involved in the coordination and, therefore, probably due to the reinstate of the conjugation among the three rings, interrupted by the metal coordination as shown in the crystal structure [42], the absorption and emission bands are restored at energies close to those of the free ligand. The lack of a full quenching of the emission and the addition of only one chloride guest suggests that the aliphatic amine function of the side arm is still involved in the coordination (data supported also by NMR data) in the chloride adduct. However, the presence of the coordinated chloride close to the PyPD acts as a quencher and the emission decreases, as schematically proposed in Figure 3.

The log *K* for the interaction between Cl^−^ and [ZnL]^2+^ was evaluated by fitting the UV-Vis and fluorescence tritation data The data showed a good fit for a 1:1 model, referring to the reaction [ZnL]^2+^ + Cl^−^ ⇌ [ZnLCl]^+^. The values obtained from the UV-Vis data (log *K* = 6.10(3)) and from the fluorescence titrations (log *K* = 6.39(6)) were in good agreement. These values highlight that the chloride anion is strongly bound by the [ZnL]^2+^ species, forming a stable adduct.

The value is higher 2–4 logarithmic units than those usually found in chloride coordination via H-bonding or charge-charge interactions [11,38,43], while it is in line, although a little bit higher, than those found for the addition of chloride to mononuclear Zn-complexes [18,37]. However, even though the influence of the solvent in the values of the formation constant is crucial, being higher in an aprotic solvent such as CH_3_CN than in water, the value found here suggests a high affinity for chloride in this system. Many reasons could contribute to this such as, for example, the coordination environment of the Zn^2+^ ion inferred by the ligand framework that might be able to exalt the affinity between Zn^2+^ and a Cl^−^ with respect to the other anions as well as the solvent and its solvation power, which might again favor the addition of chloride.

**Figure 4:**
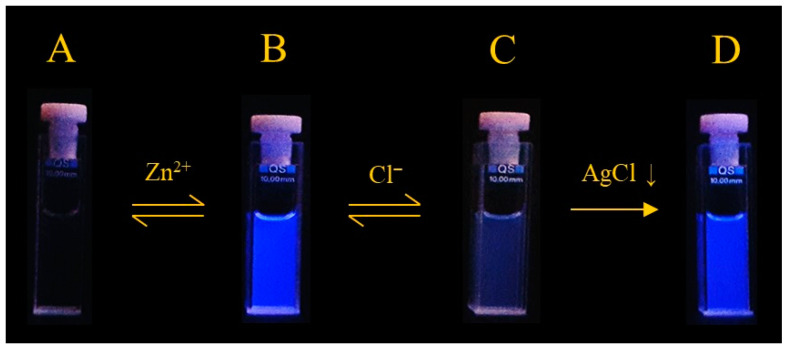
Visual emission fluorescence of solutions containing (**A**) L; (**B**) [ZnL]^2+^; (**C**) [ZnL]^2+^ + 1 equiv. Bu_4_NCl; (**D**) + 1 equiv. AgNO_3_. All solutions in CH_3_CN at 298 K, [L] = 1.35 × 10^−5^ mol·dm^−3^; images were recorded under a 365 nm UV lamp.

Interestingly, the emission is restored to that of the [ZnL]^2+^ species when adding one equivalent of AgNO_3_ to remove the chloride anion from the coordination sphere of the metal ion in the spectra, a change that can be clearly seen under a 365 nm UV lamp (Figure 4).

**Figure 5:**
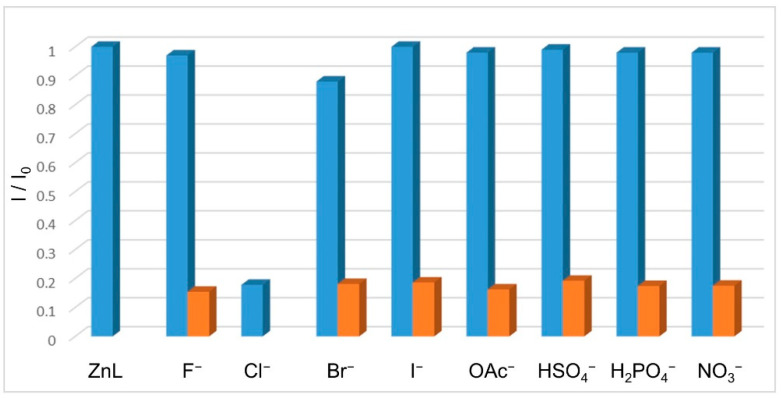
Plot of the ratio between the fluorescence emission intensity of [ZnL]^2+^ complex at 374 nm (λ_ex_ = 277 nm) (I) and the emission intensity in the presence of 1 equiv. of F^−^, Cl^−^, Br^−^, I^−^, OAc^−^, HSO_4_^−^, H_2_PO_4_^−^, and NO_3_^−^ anion (I_0_, blue bars), or in the co-presence of 1 equiv. of Cl^−^ and 2 equiv. of F^−^, Br^−^, I^−^, OAc^−^, HSO_4_^−^, H_2_PO_4_^−^, and NO_3_^−^ anions (as tetrabutylammonium salts, orange bars).

Competition experiments were also carried out by adding two equivalents of all anions studied to a CH_3_CN solution containing [ZnL]^+^ and Cl^−^ in 1:1 molar ratio (Figure 5, orange bars). The result indicates that the emission of the [ZnLCl]^+^ cation is not perturbed by the presence of other anions, highlighting the selective fluorescent response to chloride also in the presence of these possible interfering anions.

### 2.2. NMR Studies

**Figure 6:**
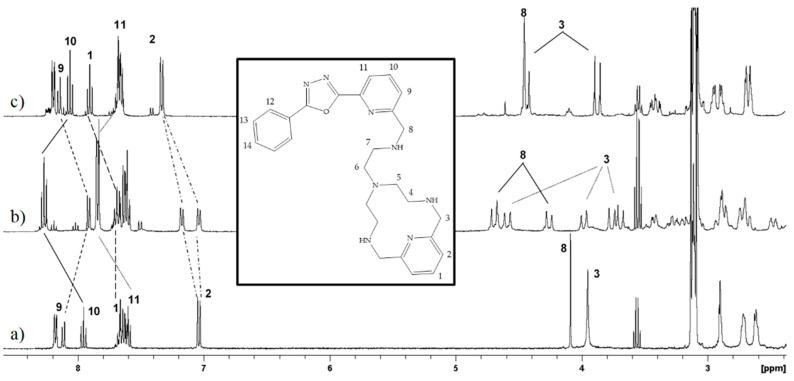
^1^H-NMR spectra of (**a**) ligand L, (**b**) ZnL complex and (**c**) ZnL complex in the presence of 1.0 equivalent of Bu_4_NCl, in CD_3_CN at 303 K. Inset: ^1^H-NMR labeling scheme.

^1^H-NMR spectra were recorded in CD_3_CN solution; the obtained spectrum for dissolving the solid [ZnL](ClO_4_)_2_ complex was the same as that obtained by adding one equivalent of Zn^2+^ to a solution containing free L. The spectrum did not undergo further modification after the addition of further equivalents of the metal ion, suggesting that the [ZnL]^2+^ species is fully formed and that it is the only one existing in solution in such experimental conditions. Figure 6 reports the spectra of the [ZnL]^2+^ species together with that of free L. ^1^H spectra recorded in D_2_O present analogous features and are collected in the ESI (Figures S4 and S5).

Comparing the spectra of free L and [ZnL]^2+^ (Figure 6a,b, respectively), the aliphatic resonances lose their equivalence on the NMR time scale due to the binding of Zn^2+^ because of the stiffening of the molecular framework produced by coordination of the metal. However, the signals of the hydrogen atoms H3 and H8 in the benzylic positions to the pyridine of the macrocycle and of the PyPD, respectively, can be safely attributed. H3 presents two distinct signals due to two AB systems (*J*_AB_ = 16.6 Hz); this means that the two methylene groups are not equivalent between them as well as the hydrogen atoms inside the same group, on the NMR time scale; H8 also exhibits an AB system (*J*_AB_ = 16.8 Hz).

While the signals of H3 shift in different downfield and upfield directions, probably because one of them is located close to the anisotropic cone of the aromatic ring, both signals of H8 shift downfield. However, these shifts can be all attributed to the involvement in the Zn^2+^ coordination of the amine functions in the benzylic position to Py and PyPD, respectively, as supported by the crystal structure of the [ZnL]^2+^ species [42].

The aromatic resonances also shift compared to those of free **L**. In particular, H1 and H2 belonging to the pyridine moiety of the macrocyclic ring shift downfield, and H2 lacks the equivalence between the two protons passing from a doublet in the free **L** in two doublets in the Zn^2+^ complex spectrum. This can be attributed to the involvement of this pyridine in the Zn^2+^ coordination. The protons H10 and H11 of the PyPD moiety also exhibit downfield shifts while H9 shifts upfield. These shifts highlight the involvement in the coordination of the pyridine of the PyPD also suggesting, together with the other pattern of signals, a similar coordination environment for the Zn^2+^ ion in solution as that retrieved in the solid state. In other words, there is evidence that in CH_3_CN solution, all amine functions of the macrocycle and the side arm, with both the pyridine rings included, are involved in the coordination of Zn^2+^.

The addition of the chloride anion perturbs the spectrum of the [ZnL]^2+^ species (see Figure 6c); the final spectrum was obtained with the addition of one equivalent of chloride and no more modifications were observed by adding further chloride (Figure S6). The change supports the binding of one chloride ion by [ZnL]^2+^, which thus behaves, as observed by spectrophotometric studies, as a selective metallo-receptor for this anion to give the [ZnLCl]^+^ species. Comparing the two spectra, the main changes are exhibited by the side arm and by the benzylic hydrogen atoms H3 and H8 (see Figure 6b,c). The resonances of the PyPD fragment return to similar chemical shifts exhibited in the free L, mainly those of H9, H10, and H11, suggesting that the binding of chloride causes the detachment of the PyPD pyridine binding group from the coordination of the metal ion and that, probably, its position is substituted by the chloride. On the contrary, H1 and H2, belonging to the pyridine of the macrocycle, shift downfield, resolving the non-equivalence of H2 signals. In the aliphatic part, a lowering of the signals due to such protons can be observed. In particular, H8 returns to a singlet with the addition of chloride while H3 shows only one AB system and, thus, two doublets; however, both H3 and H8 resonances are located in the average of the chemical shifts found in the [ZnL]^2+^ species, thus suggesting that the aliphatic amine function of the side arm remains coordinated. All these changes can be justified considering that the release of the PyPD from the coordination of Zn^2+^ and subsequent coordination of the chloride probably strengthens the bonds between the metal and the macrocyclic part and reduces the molecular stiffness. This produces a higher downfield shift of H1 and H2 and the reduction of the signals on the NMR time scale.

## 3. Materials and Methods

### 3.1. General Methods

All chemicals were purchased from Aldrich (Milano, Italy), Fluka (Milano, Italy) and Lancaster (Kandel, Germany) in the highest commercially available quality. All the solvents were dried prior to use.

### 3.2. Synthesis

Compound L, or 6-{2-[N-(6-(5-phenyl[1,3,4]oxadiazole-2-yl)-2-pyridyl)methylamino]ethyl}-3,6,9-triaza-1-(2,6)-pyridinacyclodecaphane, and the mononuclear [ZnL](ClO_4_)_2_ complex were obtained following the synthetic procedures reported in [42].

### 3.3. Spectroscopic Experiments

Fluorescence spectra were recorded at 298 K with a Varian Cary Eclipse spectrofluorometer (Agilent Technologies, Milano, Italy). UV-Vis absorption spectra were recorded at 298 K with a Varian Cary-100 spectrophotometer (Agilent Technologies, Milano, Italy) equipped with a temperature control unit. The study of the interaction of anions with ZnL complex was performed using CH_3_CN as solvent; a stock solution of ZnL was prepared adding an equimolar amount of Zn(ClO_4_)_2_ to a 1.2 × 10^−5^ mol·dm^−3^ acetonitrile solution containing L.

In a typical experiment, a solution containing the anion G (F^−^, Cl^−^, Br^−^, I^−^, NO_3_^−^, H_2_PO_4_^−^ and OAc^−^ as tetrabutylammonium salts) was added to the solution containing the metal complex, up to 5 equivalents with respect to the ligand. At least three sets of spectrophotometric titration curves for each G/ZnL system were obtained. All sets of curves were treated either as single sets or as separate entities, for each system; no significant variations were found in the values of the determined constants. The Hyperquad (2013 version) and HypSpec (2014 version, Protonic Software, Leeds, UK) computer programs were used to process the spectrophotometric data [44].

### 3.4. NMR Experiments

NMR spectra were recorded on a Bruker Avance 400 spectrometer (Bruker Italia, Milano, Italy) operating at 400.13 and 100.61 MHz for ^1^H and ^13^C, respectively, equipped with a PABBO Z-gradient direct probe and a variable temperature unit. ^1^H- and ^13^C-NMR spectra were referenced to residual solvent signals. The assignment of the NMR resonances was supported by 2D experiments (Figures S7–S9). Two-dimensional experiments (COSY, NOESY, and HSQC) were conducted using standard Bruker pulse sequences. The 2D-NOESY experiments were conducted using a mixing time (d8) of 0.35 s.

NMR titrations with anions were carried out in CD_3_CN. In a typical experiment, a 5 × 10^−2^ mol·dm^−3^ solution of the anion was added in 0.05 equiv. at a time to a 1 × 10^−2^ mol·dm^−3^ solution of the metal complex directly in the NMR tube; the tube was then kept for 5 min at a temperature of 298.1 K before starting the acquisition of the spectrum. The anions tested were added as their tetrabutylammonium salts.

## 4. Conclusions

Coordination of Zn^2+^ by the scorpiand-type azamacrocyclic ligand L, containing the pyridyl-oxadiazole-phenyl moiety, results in a sharp chelation-induced enhancement of the fluorescence. The fluorescent [ZnL]^2+^ complex can be used as metallo-receptor for secondary species as coordinating anions. The studies revealed that, among the several inorganic anions screened, only chloride is able to affect the photochemical emission and absorption properties of the metal complex. It has been shown here that the luminescent properties of the [ZnL]^2+^ complex, particularly the strong fluorescent quenching caused by the chloride anions, makes this complex a suitable optical sensor for selectively sensing chloride anions in solution.

NMR studies suggest that the recognition of chloride by the complex involves the removal of the chromophore from the coordination sphere, which explains the sharp quenching of the fluorescence observed. Since the PyPD moiety remains covalently attached to the macrocycle after the interaction, the recognition event can be described as an intramolecular fluorescence displacement assay. Moreover, this feature allows the fluorescence to be readily restored when chloride is removed from the solution.

## Supplementary Materials

The following material is available online. Figure S1: UV-Vis absorption (**a**) and fluorescence (**b**) spectra of [ZnL]^2+^ species alone and in the presence of 5 equivalents of F^−^, Cl^−^, Br^−^, I^−^, NO_3_^−^, HSO_4_^−^, H_2_PO_4_^−^, and CH_3_COO^−^ added as tetrabutylammonium salts ([L] = 1.35 × 10^−5^ mol·dm^−3^; λ_ex_ = 278 nm). Spectra recorded in CH_3_CN solution at 298 ± 0.1 K. Figure S2: UV-Vis absorption (**a**) and fluorescence (**b**) titration of [Zn**L**]^2+^ species (1.35 × 10^−5^ mol·dm^−3^; λ_ex_ = 278 nm) with Bu_4_NCl in an aqueous buffer (HEPES, 0.5 M) solution at pH = 7.4. Figure S3: UV-Vis absorption (**a**) and fluorescence (**b**) spectra of L (black line), [Zn**L**]^2+^ (red line) and [Zn**L**Cl]^+^ species (blue line) (1.35 × 10^−5^ mol·dm^−3^; λ_ex_ = 278 nm) in CH_3_CN solution at 298 ± 0.1 K. Figure S4: ^1^H-NMR spectra of L (2.0 mM) in D_2_O at pD 6.0, in the presence of increasing equivalents of Zn(ClO_4_)_2_. Figure S5: Aromatic region of the ^1^H-NMR spectra, Figure S6: ^1^H-NMR spectra of [ZnL]^2+^ (1 × 10^−2^ mol·dm^−3^) in CD_3_CN at 298 K, in the presence of increasing equivalents of Bu_4_NCl. Figure S7: ^1^H − ^1^H-COSY (**a**) and ^1^H − ^13^C HSQC (**b**) NMR spectra of L (1 × 10^−2^ mol·dm^−3^) in CD_3_CN at 298 K. Figure S8: ^1^H − ^1^H-COSY (**a**) and ^1^H − ^1^H-NOESY (**b**) NMR spectra of [Zn**L**]^2+^ (1 × 10^−2^ mol·dm^−3^) in CD_3_CN at 298 K. Figure S9: ^1^H − ^1^H-COSY (**a**), ^1^H − ^13^C-HSQC (**b**) and ^1^H − ^1^H-NOESY, and (**c**) NMR spectra of [Zn**L**Cl]^+^ (1 × 10^−2^ mol·dm^−3^) in CD_3_CN at 298 K.

## Sample Availability

Samples of the compounds are available from the authors.
