# Supplementary material for: A Metal-Based Receptor for Selective Coordination and Fluorescent Sensing of Chloride
Source: Molecules. 2021 Apr 18;26(8):2352. doi: 10.3390/molecules26082352 (PMC8073790; doi:10.3390/molecules26082352)
Supplement: Supplementary file 1 [file molecules-26-02352-s001.zip › molecules-1192429-supplementary.pdf]

Supplementary Materials

A Metal-Based Receptor for Selective Coordination and Fluorescent Sensing of Chloride

Mauro Formica [1,*], Vieri Fusi [1], Daniele Paderni [1], Gianluca Ambrosi [1], Mario Inclán [2,*], Maria Paz Clares [2], Begoña Verdejo [2] and Enrique García-España [2]

1 Department of Pure and Applied Sciences, University of Urbino "Carlo Bo", Via della Stazione 4, I-61029 Urbino, Italy

2 Institute of Molecular Sciences, University of Valencia, C/Catedrático José Beltrán 2, 46980 Paterna Valencia, Spain;

Table of Contents

Figure S1. UV-Vis absorption (a) and fluorescence (b) spectra of $[ZnL]^{2+}$ species alone and in the presence of 5 equivalents of F^-, Cl^-, Br^-, I^-, NO_3^-, HSO_4^-, $H_2PO_4^-$ and CH_3COO^- added as tetrabuthylammonium salts ([L] = 1.35×10^{-5} mol dm^{-3}; λ_{ex} = 278 nm). Spectra recorded in CH_3CN solution at 298 ± 0.1 K.

Figure S2. UV-Vis absorption (a) and fluorescence (b) titration of $[ZnL]^{2+}$ species (1.35×10^{-5} mol dm^{-3}; λ_{ex} = 278 nm) with Bu_4NCl in an aqueous buffer (HEPES, 0.5 M) solution at pH = 7.4.

Figure S3. UV-Vis absorption (a) and fluorescence (b) spectra of L (black line), $[ZnL]^{2+}$ (red line) and $[ZnLCl]^+$ species (blue line) (1.35×10^{-5} mol dm^{-3}; λ_{ex} = 278 nm) in CH_3CN solution at 298 ± 0.1 K.

Figure S4. ^{1}H NMR spectra of L (2.0 mM) in D_2O at pD 6.0, in the presence of increasing equivalents of $Zn(ClO_4)_2$.

Figure S5. Aromatic region of the ^{1}H NMR spectra.

Figure S6. ^{1}H NMR spectra of $[ZnL]^{2+}$ (1×10^{-2} mol dm^{-3}) in CD_3CN at 298 K, in the presence of increasing equivalents of Bu_4NCl.

Figure S7. ^{1}H–^{1}H COSY (a) and ^{1}H–^{13}C HSQC (b) NMR spectra of L (1×10^{-2} mol dm^{-3}) in CD_3CN at 298 K.

Figure S8. ^{1}H–^{1}H COSY (a) and ^{1}H–^{1}H NOESY (b) NMR spectra of $[ZnL]^{2+}$ (1×10^{-2} mol dm^{-3}) in CD_3CN at 298 K.

Figure S9. ^{1}H–^{1}H COSY (a), ^{1}H–^{13}C HSQC (b) and ^{1}H–^{1}H NOESY (c) NMR spectra of $[ZnLCl]^{+}$ (1×10^{-2} mol dm^{-3}) in CD_3CN at 298 K.

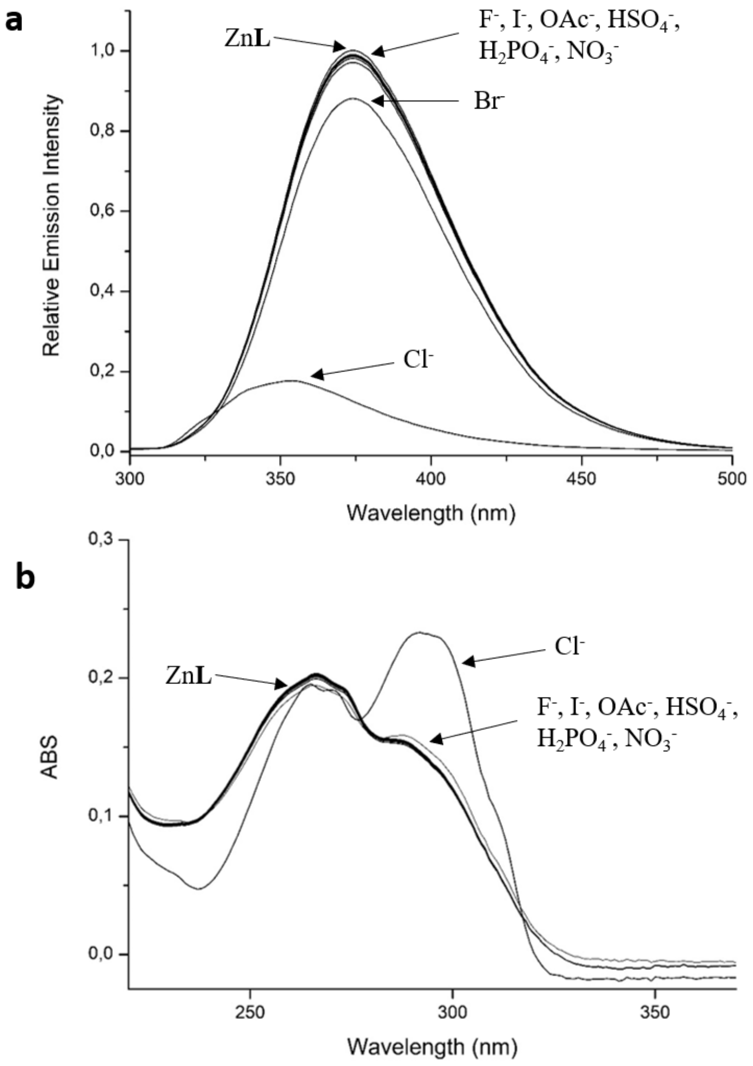

Figure S1. UV-Vis absorption (a) and fluorescence (b) spectra of $[ZnL]^{2+}$ species alone and in the presence of 5 equivalents of F^-, Cl^-, Br^-, I^-, NO_3^-, HSO_4^-, $H_2PO_4^-$ and CH_3COO^- added as tetrabuthylammonium salts ([L] = 1.35×10^{-5} mol dm^{-3}; λ_{ex} = 278 nm). Spectra recorded in CH_3CN solution at 298 ± 0.1 K.

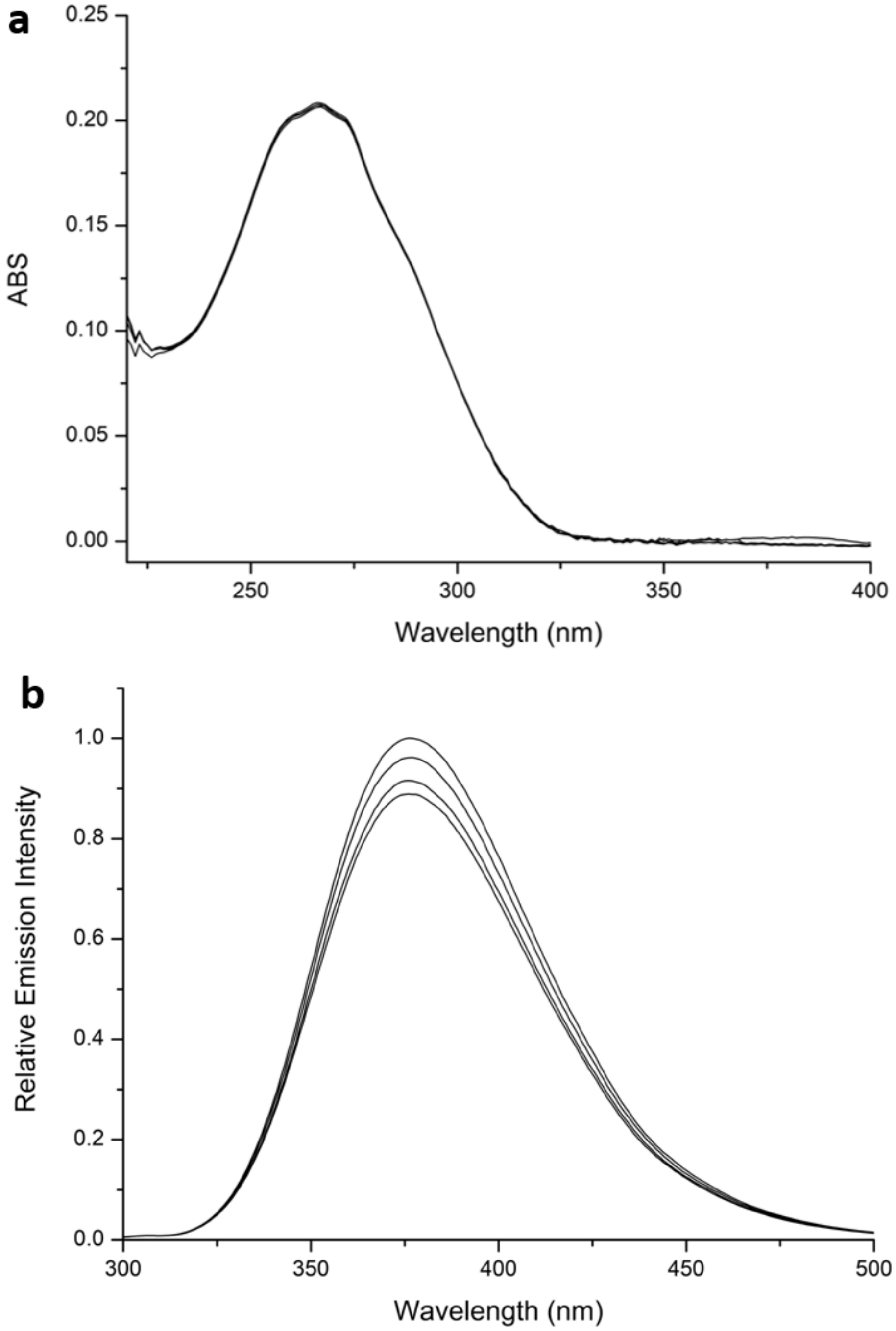

Figure S2. UV-Vis absorption (a) and fluorescence (b) titration of $[ZnL]^{2+}$ species (1.35×10^{-5} mol dm^{-3}; λ_{ex} = 278 nm) with Bu_4NCl in an aqueous buffer (HEPES, 0.5 M) solution at pH = 7.4.

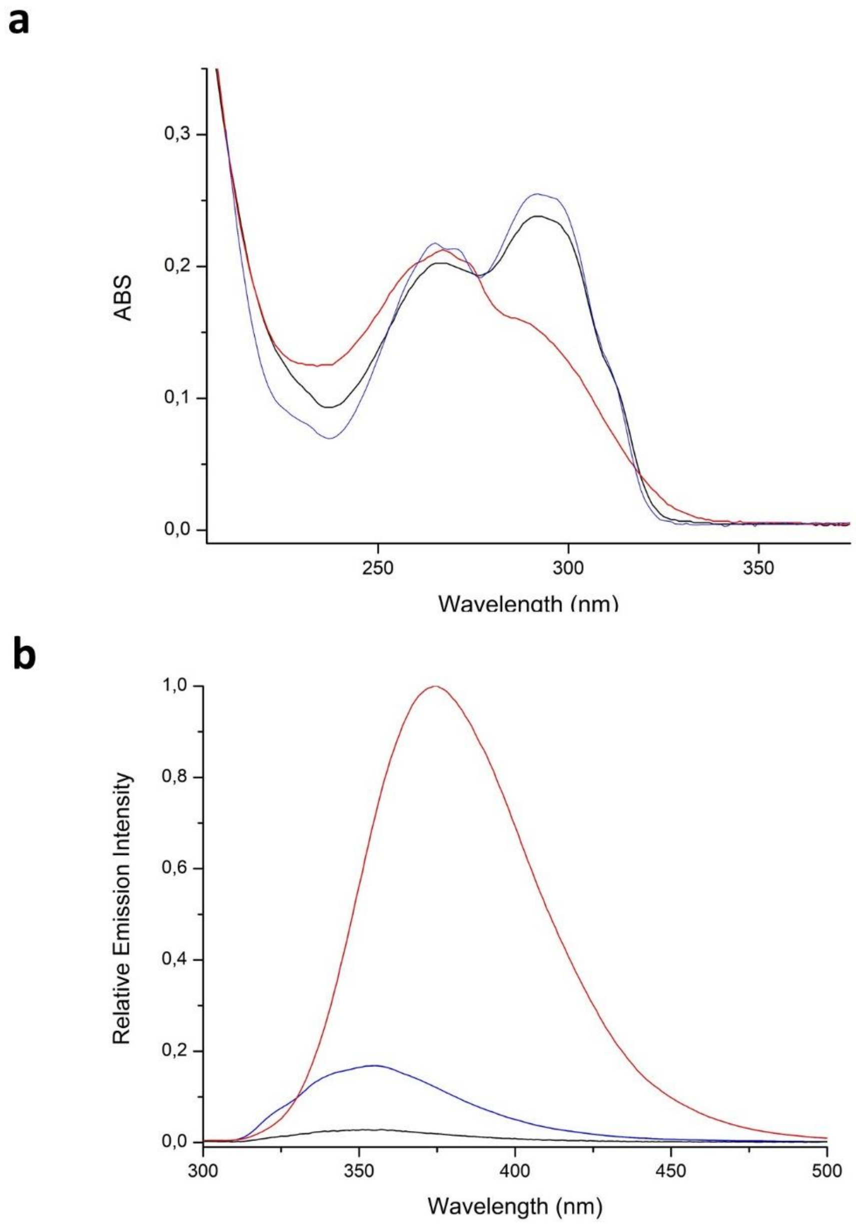

Figure S3. UV-Vis absorption (a) and fluorescence (b) spectra of L (black line), $[ZnL]^{2+}$ (red line) and $[ZnLCl]^{+}$ species (blue line) (1.35×10^{-5} mol dm^{-3}; λ_{ex} = 278 nm) in CH_3CN solution at 298 ± 0.1 K.

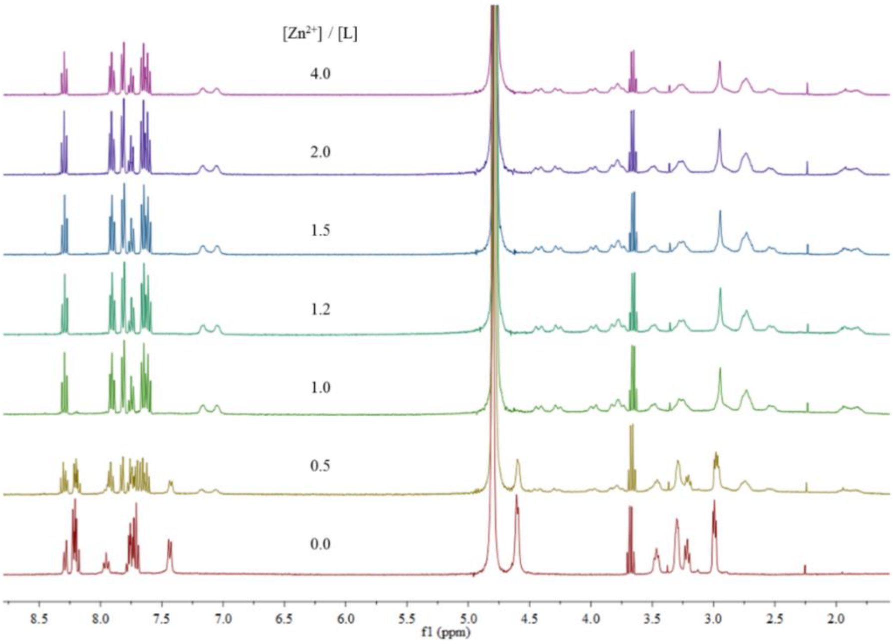

Figure S4. ^{1}H NMR spectra of L (2.0 mM) in D_2O at pD 6.0, in the presence of increasing equivalents of $Zn(ClO_4)_2$.

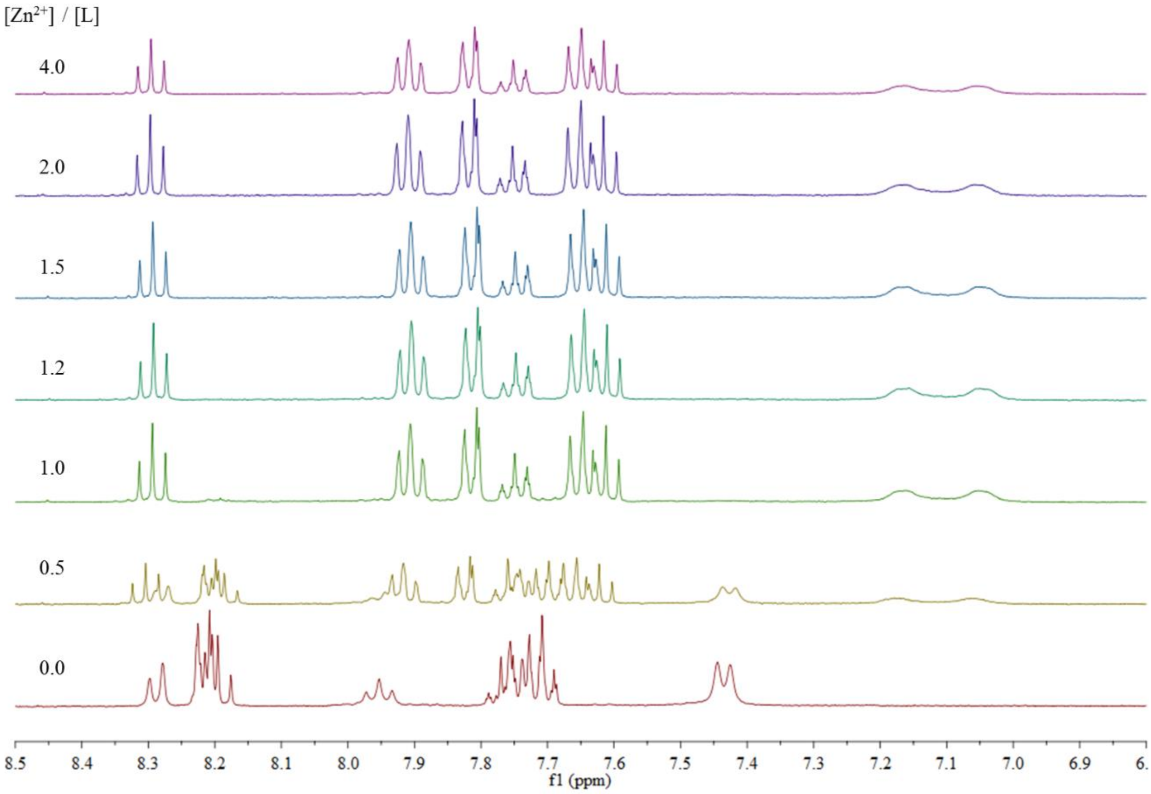

Figure S5. Aromatic region of the ^{1}H NMR spectra.

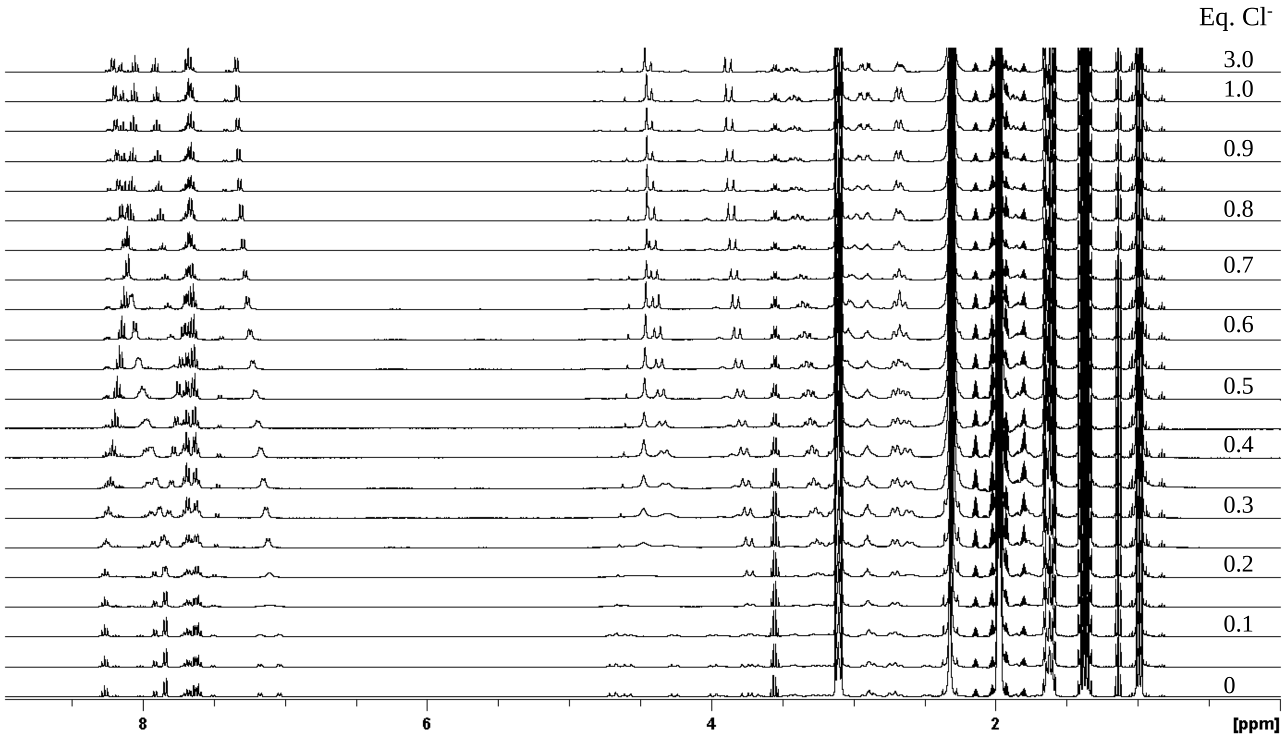

Figure S6. ^{1}H NMR spectra of $[ZnL]^{2+}$ (1 × 10^{-2} mol dm^{-3}) in CD_3CN at 298 K, in the presence of increasing equivalents of Bu_4NCl.

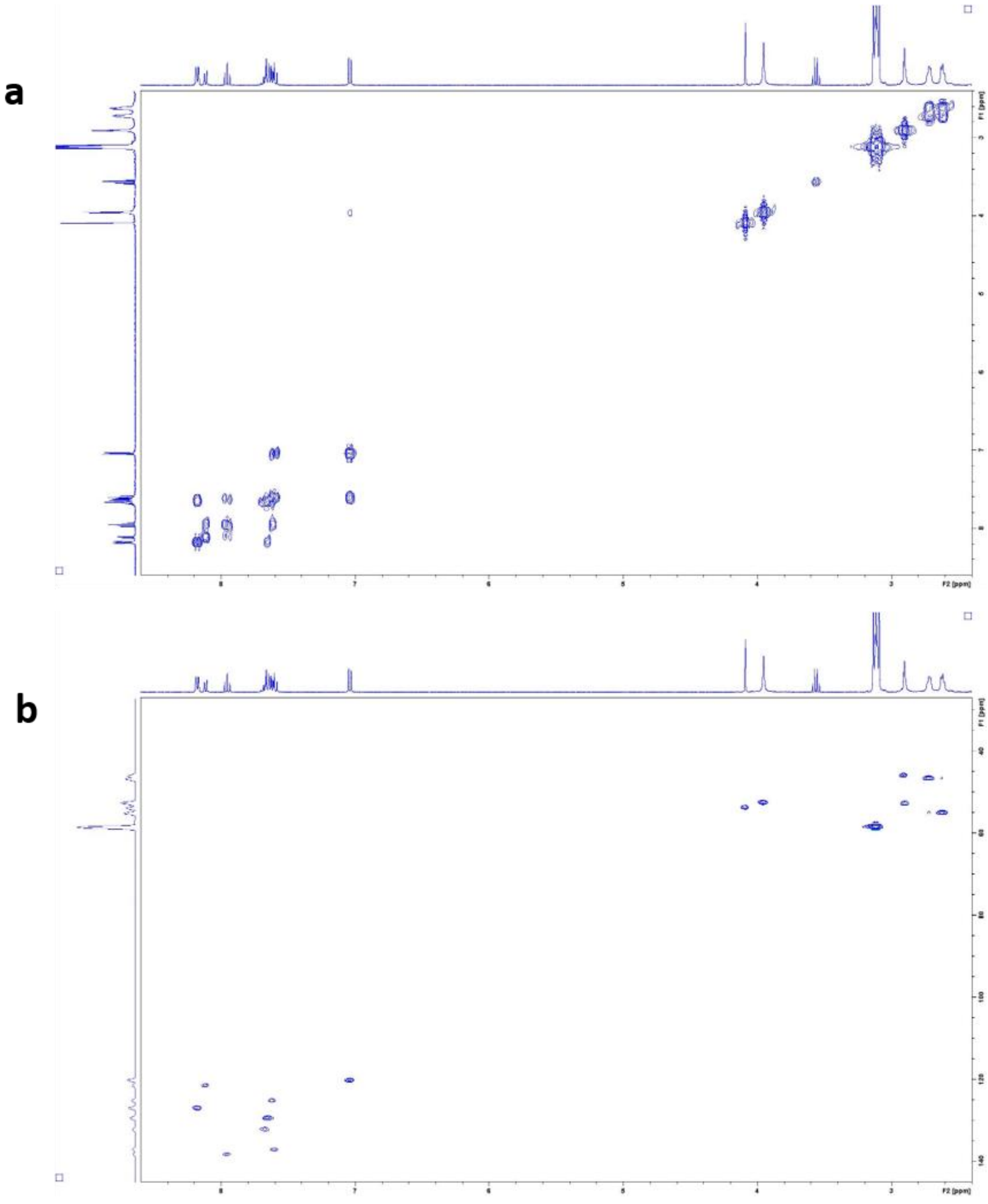

Figure S7. ^{1}H–^{1}H COSY (a) and ^{1}H–^{13}C HSQC (b) NMR spectra of L (1×10^{-2} mol dm^{-3}) in CD_3CN at 298 K.

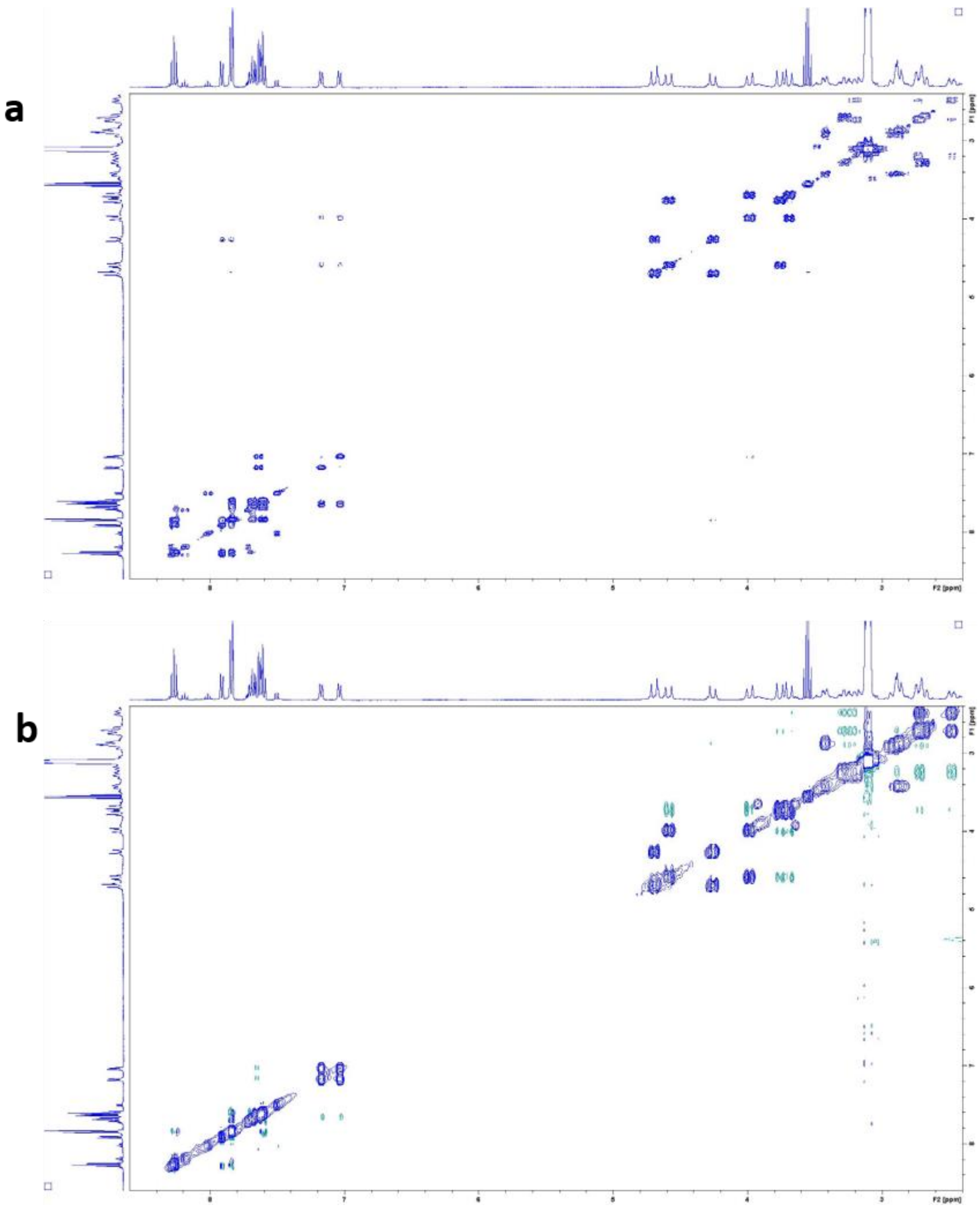

Figure S8. ^1H–^1H COSY (a) and ^1H–^1H NOESY (b) NMR spectra of $[ZnL]^{2+}$ (1×10^{-2} mol dm^{-3}) in CD_3CN at 298 K.

a

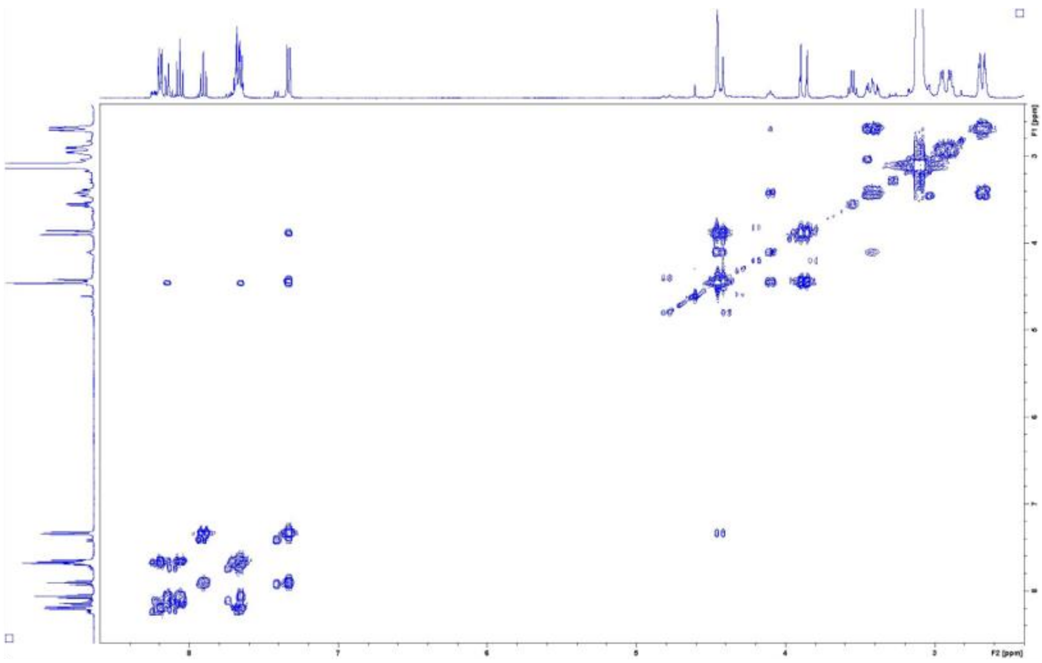

b

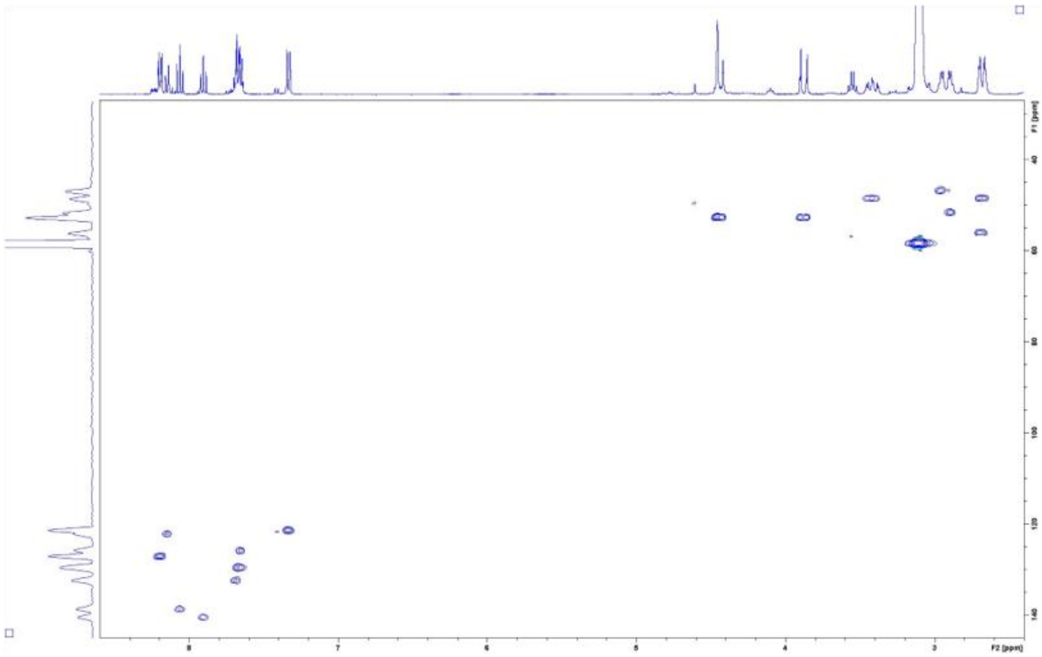

c

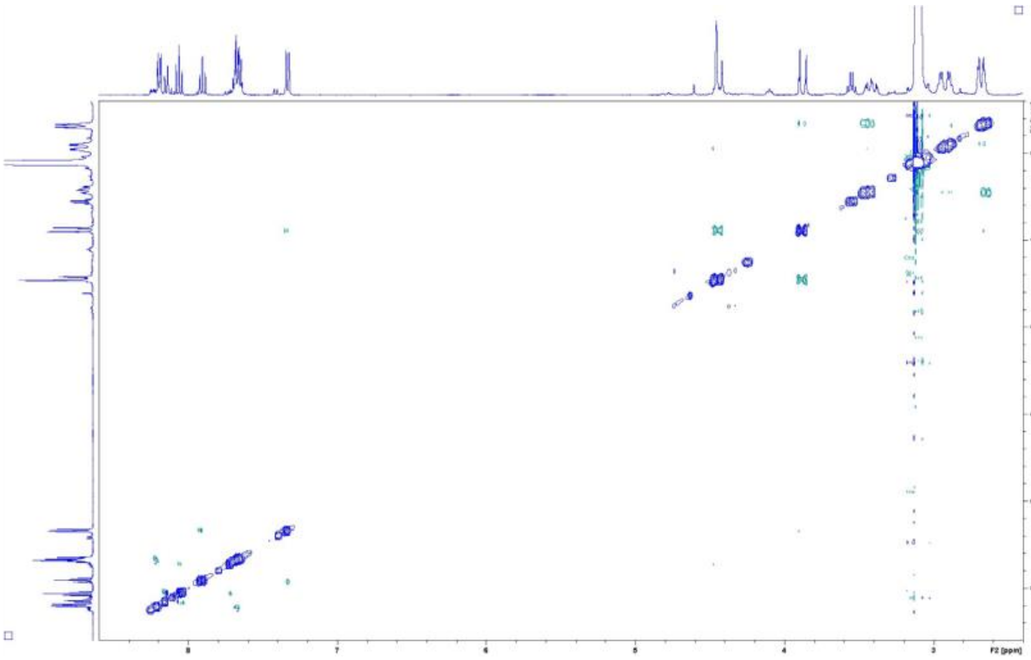

Figure S9. ^{1}H–^{1}H COSY (a), ^{1}H–^{13}C HSQC (b) and ^{1}H–^{1}H NOESY (c) NMR spectra of $[ZnLCl]^{+}$ (1×10^{-2} mol dm^{-3}) in CD_3CN at 298 K.
